# Intraoperative Frozen Section Analysis of the Pancreas: A Case Report and Review of the Literature

**DOI:** 10.1089/crpc.2016.0014

**Published:** 2016-11-01

**Authors:** Jillian W. Bonaroti, Stephen Doane, Peter A. McCue, Jordan M. Winter

**Affiliations:** ^1^ Sidney Kimmel Medical College, Thomas Jefferson University, Philadelphia, Pennsylvania.; ^2^ Department of Surgery, Thomas Jefferson University Hospital, Philadelphia, Pennsylvania.; ^3^ Department of Surgical Pathology, Thomas Jefferson University Hospital, Philadelphia, Pennsylvania.

**Keywords:** adenocarcinoma, cystadenoma, intraoperative frozen section, pancreas

## Abstract

**Background:** Intraoperative frozen section analysis is frequently used to obtain a histological diagnosis at the time of resection and to assess resection margins. Although many surgeons perceive a clinical benefit, particularly with respect to the transected resection margins, the limitations and pitfalls of frozen section analysis have not been well documented.

**Case:** Here, we report a case of serous cystadenoma with background pancreatitis masquerading on frozen section as an invasive pancreatic ductal adenocarcinoma. This interpretation was a surprise in light of preoperative imaging that was highly suggestive of a benign cystic tumor, but nevertheless prompted intraoperative consideration of a more radical operation to ensure a complete resection was achieved.

**Conclusions:** Frozen section analysis is an imperfect test, and misdiagnoses can potentially impact patient outcomes adversely. Intraoperative decisions must carefully integrate the preliminary pathological interpretation with the overall clinical context. Further studies are warranted to more fully characterize the accuracy, utility, and cost-effectiveness of intraoperative frozen section analysis for pancreatic surgery.

## Introduction

Intraoperative frozen section analysis is frequently used by pancreatic surgeons to obtain a histological diagnosis, as well as to assess resection margins. The overall usage of intraoperative frozen section is uncertain, although one recent report suggests that as many as 80% of pancreatic surgeons routinely utilize this approach.^1^ With intraoperative histological review, surgeons are able to more fully discuss intraoperative findings with patients and families upon completion of an operation. Moreover, frozen section analysis can inform intraoperative decision making. For instance, resection margins may be revised if cancer cells are identified at one of the modifiable transected margins, such as at the pancreatic neck or bile duct.

Despite the perceived advantages, this practice has been largely understudied, and the measurable benefits are unclear. Moreover, potential pitfalls are rarely discussed. Here, we report an example of an incorrect diagnosis provided by the frozen section analysis, with implications on the intraoperative surgical management.

## Case

A 71-year-old woman initially presented to an outside hospital in December 2015 with vague epigastric discomfort. A chest computed tomography (CT) was obtained to evaluate the possibility of a pulmonary embolism, and a calcified cyst was identified in the tail of the pancreas. Of note, laboratory tests were notable for a serum amylase level of 402 U/L and a lipase level of 553 U/L. The patient underwent additional imaging before surgical consultation to further characterize the pancreatic lesion, including an abdominal ultrasound, CT, and magnetic resonance imaging (MRI). The patient was then referred for a surgical evaluation.

At the time of her visit, she was asymptomatic, without abdominal pain, back pain, steatorrhea, jaundice, weight loss, or nausea. Her physical examination did not reveal any abnormalities. Pertinent laboratory data included a serum CA 19-9 level of 35 U/mL, HbA1c level of 6.1%, repeat amylase level of 378 U/L, and repeat lipase level of 439 U/L.

**FIG. 1.**
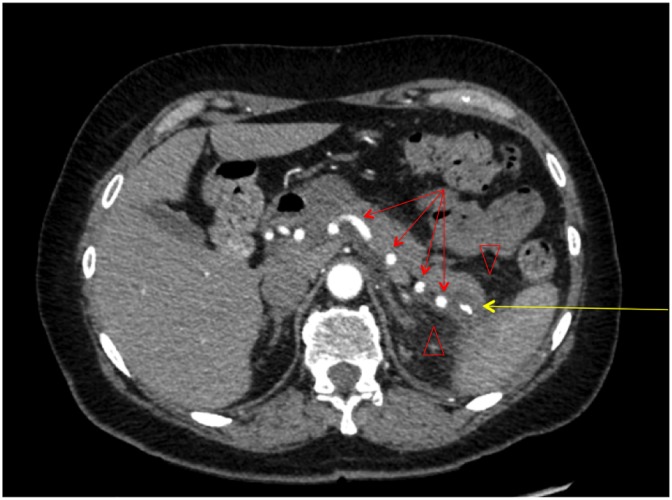
Computed tomography of the abdomen (axial view) displaying a calcified, cystic mass in the tail of the pancreas with an area of central scarring (yellow arrow) and evidence of peripancreatic inflammation and edema (red arrow heads). The splenic artery can be seen coursing through the pancreas in a tortuous manner (red arrows).

**FIG. 2.**
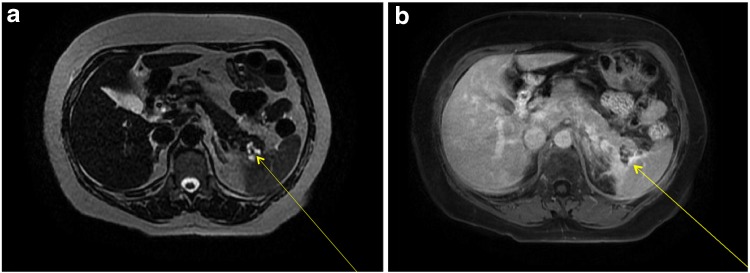
**(a)** T2 weighted single shot fast spin echo axial MRI displaying a mass in the pancreatic tail (yellow arrow) **(b)** Axial LAVA 5 min delay MRI displaying presence of a cystic mass in the pancreatic tail and peripheral postenhancement (yellow arrow). MRI, magnetic resonance imaging.

A review of her abdominal imaging revealed a 3 cm complex cystic mass in the pancreatic tail, with thickened septa, and an associated solid nodule that appeared calcified, and was associated with central scarring (Fig. 1). These findings were suggestive of a benign serous cystadenoma. Peripheral enhancement was observed on an MRI (Fig. 2a, b), raising the possibility of a mucinous cystic neoplasm as well. Peripancreatic edema on CT (Fig. 1) was interpreted as evidence of acute pancreatitis, further confounding the clinical picture. Endoscopic ultrasound and aspiration of the cyst were not performed because of anatomic inaccessibility related to the cyst's location near the splenic hilum, and away from the posterior wall of the stomach. The patient was offered resectional therapy based on the fact that her symptoms and serum tests reflected pancreatitis, attributable to the pancreatic cyst. The possibility of a mucinous neoplasm and a serum CA 19-9 level at the upper range of normal also factored into the decision.

**FIG. 3.**
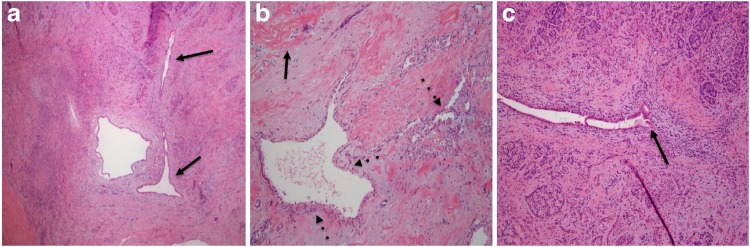
Frozen section pathology sections displaying **(a)** irregular ductal structures (solid arrows), **(b)** diffuse desmoplastic and sclerotic stroma (solid arrow), and atypical, infiltrating epithelium (dashed arrows), **(c)** compressed and incomplete ductal lumen, evident by discontinuity in ductal epithelium (solid arrow). (All hematoxilin–eosin stained).

A laparoscopic distal pancreatectomy with *en bloc* splenectomy was performed without incident, although the dissection was challenged by the peripancreatic inflammation observed on imaging, which obscured the natural plane between the splenic vessels and the pancreas. On gross inspection of the resected specimen, the lesion had the appearance of a solid mass with infiltrating tentacles radiating toward the distal resection margin. A cystic component was not immediately apparent. Microscopic examination revealed desmoplastic stroma, which distorted the glands, and atypical epithelium (Fig. 3a–c) infiltrating toward the edge of the resection margin. These findings were interpreted to be consistent with invasive ductal adenocarcinoma, and background chronic pancreatitis. The proximal pancreatic neck margin was believed to be microscopically negative for invasive cancer. However, on gross inspection, abnormal tissue extended up to the transected parenchyma, leaving a possibility for a revised diagnosis of a positive resection margin on final pathological review.

In light of these unexpected results, the surgical team contemplated converting to an open operation to resect additional parenchyma, and ensure a safe and complete resection in the context of pancreatitis, and a difficult laparoscopic dissection. However, after discussing with the family that the frozen section review was discordant with preoperative imaging, the decision was made to terminate the procedure, close the laparoscopic port site incisions, and defer any further management decisions regarding a more extensive resection until the final pathological review was finalized. Parenthetically, the intraoperative pathological review and family discussion added roughly 30 min to the operation. The postoperative course was uneventful, and the patient was discharged on the third postoperative day. At 6 months follow-up, the patient is well.

**FIG. 4.**
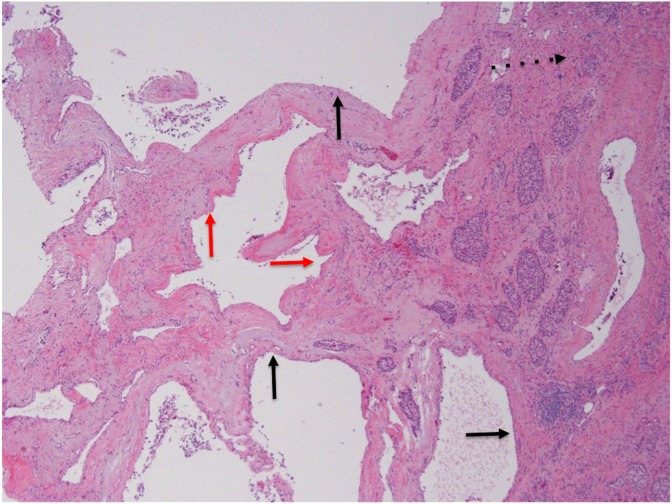
Permanent pathology specimen displaying simple cystic epithelium (solid black arrows) of a calcified microcystic serous cystadenoma. Apparent distortion in simple cystic morphology (red arrows) is caused by mass compression and not dysplasia. Background of chronic pancreatitis made evident by atrophied acinar tissue (dashed arrow, upper right corner).

In the final analysis, the lesion was determined to be a calcified microcystic serous cystadenoma with background chronic fibrosing pancreatitis. There was no evidence of invasive adenocarcinoma (Fig. 4).

## Discussion

Intraoperative frozen section analysis is frequently used to confirm preoperative diagnoses of pancreatic lesions and ensure negative resection margins. As an example of the potential value of frozen section analysis, one study demonstrated that the long-term survival after completion of total pancreatectomy for pancreatic ductal adenocarcinoma was found to be longer than patients undergoing partial pancreatectomy with positive resection margins.^2^ Moreover, frozen section analysis permits a more complete discussion of the intraoperative findings with patients and their families immediately after the operation.

However, intraoperative frozen section analysis may have limitations that could complicate patient management, although this has not been explored in any depth in the pancreatic surgery literature. For instance, it is possible to imagine undesired results stemming from a misdiagnosis. In the case presented herein, a microcystic serous cystadenoma in the background of chronic fibrosing pancreatitis masqueraded as invasive ductal adenocarcinoma on frozen section analysis, and the correct interpretation was only clearly appreciated on the final pathological review. It is likely that ductal compression from the weight of the cyst itself, along with fibrosing architecture related to pancreatic inflammation, contributed to the false appearance of abnormal ducts surrounded by peritumoral stroma. Lechago has reported that fibrosis, atrophy of exocrine structures, and formation of reactive pancreatic ductal structures in chronic pancreatitis make it difficult to distinguish between benign and malignant pathologies.^3^ The challenge posed by this scenario for frozen section analysis has been echoed by others.^4,5^

A retrospective study published in 2013 by Nelson et al. characterized the accuracy of frozen section analysis and its impact on outcomes in 68 patients undergoing pancreatic resection.^6^ The authors compared the frozen section analysis with final permanent pathology, and assessed the accuracy of histological diagnosis and the resection margins. They determined the overall accuracy for final margin status to be 97% on frozen section. However, the histological diagnostic accuracy was just 83%. The authors did not identify any false positives, unlike the present case. Similar studies have reported relatively low false positive rates ranging from 0.0 to 3.0%.^4,7–9^ A contemporary, larger scale study may be beneficial to achieve a comprehensive understanding of the clinical utility of frozen section analysis in the modern surgical era, particularly with increased use of neoadjuvant therapy.

## Conclusion

Intraoperative frozen section analysis is routinely performed and may have benefit in the surgical management of pancreatic disease. However, pathological review during the operation has limitations and can potentially be misleading. In particular, chronic pancreatitis can masquerade as invasive ductal adenocarcinoma. Intraoperative decisions should carefully integrate the frozen section analysis with other intraoperative and preoperative data. Additional studies on the utility, impact, and cost-effectiveness of intraoperative frozen section analysis after pancreatectomy are warranted.

## Abbreviations Used

CT: computed tomography
MRI: magnetic resonance imaging
